# Beyond knowledge and skills: the use of a Delphi study to develop a technology-mediated teaching strategy

**DOI:** 10.1186/1472-6920-13-51

**Published:** 2013-04-10

**Authors:** Michael Rowe, Jose Frantz, Vivienne Bozalek

**Affiliations:** 1 Department of Physiotherapy, University of the Western Cape, Cape Town, South Africa; 2 Directorate of Teaching and Learning, University of the Western Cape, Cape Town, South Africa

**Keywords:** Knowledge and skills, Blended learning, Clinical education, Professional attributes, Teaching and learning, Technology-mediated instruction

## Abstract

**Background:**

While there is evidence to suggest that teaching practices in clinical education should include activities that more accurately reflect the real world, many educators base their teaching on transmission models that encourage the rote learning of knowledge and technical skills. Technology-mediated instruction may facilitate the development of professional attributes that go beyond “having” knowledge and skills, but there is limited evidence for how to integrate technology into these innovative teaching approaches.

**Methods:**

This study used a modified Delphi method to help identify the professional attributes of capable practitioners, the approaches to teaching that may facilitate the development of these attributes, and finally, how technology could be integrated with those teaching strategies in order to develop capable practitioners. Open-ended questions were used to gather data from three different expert panels, and results were thematically analysed.

**Results:**

Clinical educators should not view knowledge, skills and attitudes as a set of products of learning, but rather as a set of attributes that are developed during a learning process. Participants highlighted the importance of continuing personal and professional development that emphasised the role of values and emotional response to the clinical context. To develop these attributes, clinical educators should use teaching activities that are learner-centred, interactive, integrated, reflective and that promote engagement. When technology-mediated teaching activities are considered, they should promote the discussion of clinical encounters, facilitate the sharing of resources and experiences, encourage reflection on the learning process and be used to access content outside the classroom. In addition, educational outcomes must drive the integration of technology into teaching practice, rather than the features of the technology.

**Conclusions:**

There is a need for a cultural change in clinical education, in which those involved with the professional training of healthcare professionals perceive teaching as more than the transmission of knowledge and technical skills. Process-oriented teaching practices that integrate technology as part of a carefully designed curriculum may have the potential to facilitate the development of capable healthcare graduates who are able to navigate the complexity of health systems and patient management in ways that go beyond the application of knowledge and skills.

## Background

Effective clinical practice requires that health professionals work within the dynamic, non-linear and complex environments of healthcare systems, and to engage with ill-structured problems that have no clear solutions [1]. They need to “adapt to change, generate new knowledge, and continue to improve their performance” over time. These attributes (defined as *capability*) [2] require more from the practitioner than a mere set of knowledge and technical skills. In order to effectively operate within the complex environments of healthcare settings, practitioners need abilities that include, but go beyond the knowledge and basic technical skills (defined as *competence*) [2] that are emphasised in undergraduate training. This includes having positive attitudes towards continuing professional development, lifelong learning, evidence-based practice, information and knowledge management and interprofessional collaboration [2].

In addition to discipline-specific knowledge, technical skills and generic attributes, healthcare practitioners are also moral agents who make decisions about patients based on personal connections and relationships with them. Values, beliefs and emotional factors are embedded within the interactions between healthcare providers and patients, suggesting that these interactions are more than the exchange of information. This active engagement with, and acknowledgement of, the emotional response to patients’ stories can help to develop the moral agency that is a necessary part of ethical clinical practice [3].

However, developing these attributes and attitudes requires a cultural change in teaching practices that focus on the development of knowledge and skills. Many clinical educators still adhere to a lecture and transmission-based approach to teaching [4], which is problematic if capability is the goal because it cannot be passively assimilated, and requires significant changes in clinical education that move it from being product- to process-oriented [2]. Teachers who adopt a transmission-based approach to teaching encourage the rote learning of facts, and a resultant superficial understanding of the topic. In contrast, teaching approaches that focus on the process of conceptual change lead to deep learning [4], and include informal and unplanned, self-directed and non-linear learning experiences. Specific strategies include, among others, experiential learning, reflective exercises, feedback, peer-supported small groups, case-based and problem-based learning, and role play [5].

Some clinical educators are beginning to experiment with technology-mediated teaching and learning practices, which blends classroom-based, face-to-face learning experiences with online interaction. This approach creates alternative means of communication between teachers and students, as well as deeper and more meaningful engagement with media-rich content. But, blended learning goes beyond the addition of technological components and requires a “…rethinking and redesigning [of] the teaching and learning relationship” [6,7]. If the integration of technology into the curriculum is to be effective, it must move beyond content transmission and aim to facilitate communication and reflection in teaching and learning practices that are interactive, flexible, collaborative and authentic [8].

There are limited studies on the development and implementation of blended learning strategies within clinical education [7], with some authors asserting that “…the current pedagogic evidence base about these tools in the context of medical/health education is seriously lacking” [9]. This presents a challenge. Technological components cannot simply be tacked on to traditional approaches without careful consideration [10] but clinicians are usually not course designers, and neither of them are necessarily educational technologists. The different stakeholders may therefore lack the diverse skills necessary to effectively integrate technology into a blended curriculum that aims to develop the attributes required for effective clinical practice. Without a sound evidence base to work from, technology-mediated instruction in clinical education may be implemented without the necessary preparation and design. In order to prepare healthcare students for the dynamic and complex clinical environment, how can we ensure that technology-mediated instruction facilitates the development of both competent and capable practitioners?

In order to address these challenges, this study used a modified Delphi approach to identify technology-mediated teaching strategies that aim to develop capability in undergraduate healthcare students. The study is significant in that it identifies attributes that go beyond knowledge and skills, as well as strategies that could be used to develop those attributes within a technology-mediated approach to teaching and learning.

## Methods

### Research design

The Delphi method is a research design that usually involves three rounds of surveys that are distributed to a panel of experts, with each round being informed by responses to the previous one. Delphi studies are used most often to gather data from domain experts with the intention of coming to consensus, often around poorly defined topics, such as developing programme alternatives [11]. There are no criteria upon which to determine the nature of the “expert”, the optimal panel size or even selection criteria of the panelists in a Delphi study [12].

The Delphi method has been used to determine the desirable attributes of physiotherapy students [13], to identify the key performance areas and assessment criteria for clinical performance among undergraduate physiotherapy students [14], and to determine competency in teaching practices [15]. It was therefore felt that a Delphi study was appropriate for this study, as it has been demonstrated to be effective in similar areas. However, this study used a modified version of the traditional Delphi, in which a different panel of experts was consulted in each round, in order to gain insight into the different challenges with technology integration that have been highlighted. Although the panels were different for each of the three rounds there was significant overlap between the panel members in the first two rounds.

The questions for the first round were based on a review of the relevant literature, with those for each subsequent round being derived from the previous responses. While a traditional Delphi study only uses open-ended questions in the first round, this study used them in each of the three rounds. In addition, since our objectives were not to reach consensus, the statistical analyses for rating participant responses were also excluded, and responses from each round were analysed qualitatively [11].

### Panel participants

The panels for the study were purposively selected from within the researcher’s personal and professional networks of practice, and included both South African and international experts. Table 1 below presents information to support the panel selection.

**Table 1:** Participants’ professions and experience in each round of the study

**Demographic information**	***Round 1***	***Round 2***	***Round 3***
Number of participants	25	21	13
Occupation*			
- Professor	7	3	2
- Lecturer	9	4	3
- Clinician	6	3	1
- Other	8	11	0
Profession			
- Physiotherapist	11	8	3
- Physician	7	3	3
- Surgeon	2	0	0
- Nurse	2	0	0
- Other	0	2	0
Years of experience			
- Range	2–36	4–25	15–25
- Average	19	14	21
Highest degree obtained			
- BSc	4	4	0
- MSc	12	3	3
- PhD	4	4	3
- M. Med	4	1	0
- Post Doctorate	1	0	0
Additional qualifications			
- Educational	13	4	6
- Clinical	11	11	3
- Management	1	0	0
- Other	0	1	1

* Note: Participants did not complete all sections of the questionnaires, hence the totals are inconsistent.

The first round sought to answer the question: “What do we want our healthcare graduates to be, as opposed to what we want them to do?” The aim of this round was for clinicians and clinical supervisors to identify the attributes of competent and capable healthcare professionals. The panel for the second round were guided by the question: “What teaching strategies would you use in order to develop the attributes identified in the first round?” The third round of the study, the focus of this paper, was guided by the question: “What are the ways in which technology-mediated instruction can be used to support the teaching strategies identified in round two?” The panel for this round included educational technologists and clinical educators with experience in integrating technology into teaching practice.

### Procedure

The three rounds of surveys ran from October, 2011 to March, 2012. Questionnaires were sent to participants by email, or they were able to complete each round using an online, web-based survey. Emails were sent using the “Blind Carbon Copy” (BCC) feature of email, so that none of the recipients were able to see who the other panel members were. Reminders were sent out two weeks after the initial surveys were emailed. Round one was sent out in October and the results were analysed in November. These results led to the development of the second round survey, which was sent in December. The results of the second round were analysed in January, and led to the development of the third survey, which was sent in February. These final results were analysed in March, 2012.

### Data analysis

The surveys consisted mainly of open-ended questions and responses were therefore analysed qualitatively. Participant responses were analysed thematically until saturation - the point at which no further themes were derived - was reached [16]. These emergent themes were then summarised and used to derive the questions for the next round of the study. Trustworthiness of the analysis was established using a framework for qualitative research that identifies the following criteria against which to judge the work; credibility, transferability and dependability [17]. The analysis, emergent themes and subsequent surveys were cross-checked by two other researchers who provided critical input on the results and analysis. In addition, the results are presented as quotes from the original text, and serve as supporting evidence for the themes that arose which, together with the critical review of two independent researchers, serves to establish both the credibility and dependability of the claims. The transferability of the claims is limited considering the specific context in which this study took place. However, transferability might be feasible depending on the similarity of other contexts to this one.

### Ethical considerations

The study received ethical clearance from the University of the Western Cape Ethics Committee (project registration number: 09/8/16). All panelists received an information sheet for each round of the study and were asked to explicitly state their consent to participate. Panelists were not required to participate in this research project and non-participation had no negative effects on those who were invited. They could withdraw from the study at any stage, and have their responses removed from the database. All responses were anonymous. Panelists who chose to participate in the first round were under no obligation to participate in subsequent rounds.

## Results

The major findings of the study are presented as responses to the overarching question that informed the previous round. However, only summaries of the first two rounds are presented here, in order to contextualise the findings of the third round. Responses from the third round are presented here in aggregate, as a narrative of the themes that arose during the analysis, and supporting quotes are presented.

Panelists in the first round were asked to identify what they thought capable students should “be”, as opposed to what they were expected “to do”. They strongly emphasised a process of active engagement with people and concepts when it came to the characteristics of “being” a professional. They spoke of students needing to engage with and be willing to be part of a developmental *process*, in addition to “having” knowledge, skills, understanding and attitudes, which were seen as *products* or final outcomes of a competent student. They also emphasised the personal, affective components of students’ approaches to practice, taking into account the challenges that they often face, and giving voice to the complexity of the clinical context. Panelists identified the challenges of authentic engagement with ethical contexts in healthcare, again highlighting the complexity of the situations that students face and the emotional context in which healthcare is practised.

In the second round, clinical educators were asked how they would go about facilitating a developmental learning process rather than focusing on the products of learning. Many panelists reported combinations of teaching strategies instead of only one approach. Teachers should provide a safe space for students to explore the domain independently, rather than telling students what they need to know. For this, appropriate role modeling is important, in which teachers demonstrate to students not only what to do and to know, but how to be. Using paper patients in small group sessions with guided discussion was a common suggestion, especially around the development of clinical reasoning and critical thinking. Educators should encourage the sharing of personal values and experiences among students and clinicians, as well as the impact of those experiences on themselves. They should build reflective components into the curriculum, asking students how they deal with stress and emotion, and then how they feel about, and deal with, those responses. Students should be encouraged to provide evidence of engagement with their own emotional responses through reflective self-report, which should include a feedback component from peers and more experienced clinicians, who each provide alternative viewpoints. They should also be encouraged to develop agency and active engagement with each other, rather than being passive recipients of information.

In the third round, which will now be focused on in detail, educational technologists and clinical educators were asked about the use of technology in teaching and learning contexts that supported continuing professional development, knowledge and skills acquisition, and emotional responses to clinical practice. Participants described a range of technology-mediated teaching practices that were interactive, integrative and reflective in nature, and which made use of technological features that enhanced student-centred and self-directed learning. In terms of using technology-mediated teaching practices to facilitate the development of lifelong learning and continuing professional development, participants reported that ICTs (Information and Communication Technology) offered a more flexible approach to learning. However, participants also suggested that underlying personal motivation and attitudes were more important than specific technological tools.

“[technology] can be exploited to encourage sharing, debate, questioning and thought provocation. Experts can role model the behaviours by posting links to recent research, plus corresponding questions to encourage further discovery and discussion.”

“The promotion of self-regulation is important in health-professionals education because it underpins the principles of PPD^a^ or lifelong learning, as well as non-technical skills development. ICT may be used to develop self-regulation skills as long as the technology is designed around the teaching and not the other way around.”

“Personal and professional development, i.e. lifelong learning is dependent on the personal attitudes and behaviour of an individual. No ICT per se has the ability to develop the attitudes and values which underpin the principles of lifelong learning. Nevertheless, ICTs may help facilitate PPD as certain professional…organisations have shown. [However], the role of ICT in PPD is secondary to the greater problem of self-assessment and self-regulation amongst healthcare professionals.”

In terms of using ICTs to develop knowledge and skills in the clinical context, participants suggested a range of strategies that promoted interaction, reflection and self-directed learning. In addition, participants advocated the use of ICTs to create more integrated learning experiences that went beyond merely learning facts. The following quotes are presented in support of these ideas:

“Communities of practice are groups of people who share a concern or a passion for something do and learn how to do it better as they interact regularly. ICTs offer greater opportunities for people to create such communities and engage in a ‘process of collective learning in a shared domain of human endeavour’.”

“Reflection can be personal or interpersonal activity, therefore ICTs which foster learning alone or with others may be suited for this purpose. Blogs or even forms of social media which require learners to analyse, evaluate or create knowledge may facilitate reflection-in-action or on-action…[Virtual patients] may allow learners to analyse, evaluate and create new knowledge, whereas learners may be limited as to how much knowledge they can reliably demonstrate using paper-based activities.”

“ICT can be used to promote engagement and interactivity. Audience response systems (ARS) come to mind as a method for facilitating this aim. The same may be true in the context of practical demonstrations. Learners can give feedback about performance during a practical demonstration.”

One of the main themes that emerged was the use of technology to displace content in time and place, moving it out of the classroom in order to create space for discussion and engagement. One common suggestion was for teachers and students to make use of technology to record practical demonstrations and lectures, thereby shifting the content to be available anywhere, anytime.

“Lectures can be provided as audio/video for the student to consume prior to meeting face-to-face (ie flipped classroom).^b^ The face-to-face component can then be devoted to rich learning experiences such as demonstrations, role plays and Q&A’s.”

“ICTs should be considered the foundation stone of clinical study. Relevant tools and resources empower the students to direct their own learning, according to a predefined program or curriculum. Face-to-face sessions can then be focused on enriching and extending the learning experience and making it authentic.”

“Lectures could be recorded and made available to students via a virtual learning environment (VLE) or other institutional platform to view online or for download to student devices. Videos/podcasts of procedures of clinical skills could also be made for students to download and support just-in-time learning either via VLE or iTunes-U.”

In terms of using technology to help support students’ emotional responses to complex clinical situations, participants’ suggested that it be used to create both synchronous and asynchronous supportive environments in which students could share difficult clinical encounters, and discuss those situations in a safe space. The sharing of experience should come from both teachers and students, as appropriate responses to ethical challenges could therefore be modelled to students.

“Creating a space where students can share their experiences and feelings without feeling threatened or judged: a simple example: the inbox message space of [social networks] allows students to share their experience with someone they trust and with whom they can be honest and open without feeling judged.”

“Supporting students’ values and emotional responses may be facilitated by ICTs, especially through the use of blogs or discussion forums.”

“drawings, poems, music to reflect moods and feelings with discussion on blogs and/forums to unpack the ‘art work/drama’.”

While the use of technology to support the sharing and discussion of students’ emotional responses to clinical situations was encouraged, several participants cautioned against the idea that technology is the best way to engage with students around sensitive topics. They suggested that working with students face-to-face at the moment of (or soon after) the clinical encounter is generally more appropriate.

“…this is one area where I think that personal contact with a senior doctor is essential. This is particularly true after traumatic incidents such as when the student participates in a resuscitation and the patient dies, or when they have a needle stick injury from an HIV + patient.”

“I think that f2f^c^ is definitely the safest way to get this kind of feedback. Usually ICT makes it harder for us to get cues that we normally use when giving or getting feedback. So with sensitive areas then we need to be especially careful.”

“I would prefer discussion to occur synchronously alongside or immediately after a learning encounter, however ICT may facilitate discussion to continue asynchronously after the learning activity is completed.”

Finally, while participants described the role of technology in teaching practices as being positive, they also suggested caution, in the sense that “the teaching should drive the technology, and not the other way around”. The following quotes are suggestive of a considered approach to the integration of technology into the curriculum.

“…the role of ICT is secondary to the environment in which the learning or reflection occurs.”

“Print, broadcast media, computers and diffused networks have introduced at least 4 new layers of mediation. It is often the affordances of these mediation layers that capture the attention of teachers and not their students pedagogical needs. When this happens, teaching suffers. When learning, however is foregrounded, and demands of pedagogy & subject matter come before bells and whistles, then technology can indeed enhance and enrich the teaching and learning process.”

“ICTs can be extremely effective at bringing together learning from across a curriculum. This requires skilled instructional design, rather than technology per se.”

## Discussion

This study highlights several themes that are relevant for those interested in using blended learning as part of clinical education. These are summarised as follows: personal and professional development must go beyond “having” knowledge and skills, and should incorporate students’ emotional responses and personal values; clinical educators who aim to develop these attributes should consider teaching practices that are interactive, integrated, reflective and formative; technology-mediated teaching and learning can facilitate the development of attributes that have an impact on professional practice; and integrating technology into teaching practice goes beyond simply choosing what tools to add to the curriculum. This discussion will emphasise the findings of the third round of the study, which looked at technology-mediated instruction, although a brief summary of the first two rounds are presented to provide context.

The first round of the study identified the attributes of healthcare practitioners that went beyond simply having knowledge and technical skills. These attributes were described by participants in terms of a *state of being*. In a world where what you “know” is quickly outdated, a sense of self that enables students to adapt to dynamic conditions is essential. By cultivating a sense of *being* rather than *knowing*, the curriculum becomes “future-proof” [18]. This process-centred notion acknowledges that knowledge should not be perceived as a static, linear set of facts. Instead, by considering it as dynamic, non-linear and multidimensional, we can help students prepare for the complexity of clinical practice by making use of teaching practices that facilitate the development of capability [2].

In the second round of the study, clinical educators advocated a combination of different approaches that sought to develop more complex outcomes than merely the ability to perform a procedure, or know a fact. The developmental nature of the process was emphasised, highlighting the importance of feedback and formative assessment as part of the process, rather than a separate function. These integrated teaching and learning practices emphasised the connection and interaction between people in a process that “values human relationships” [19]. It seems that many of the teaching strategies suggested in this study are a response to “the urge to reach forward to newer, more interactive, authentic, integrative and transformative approaches to learning and teaching” [20]. There is evidence to suggest that the instructivist paradigm of “transmitting” knowledge from teacher to learner must give way to constructivist models that facilitate the social nature of teaching and learning [2].

Constructivist approaches to teaching and learning have been demonstrated to be enhanced through the use of technology-mediated instruction, particularly when thoughtfully implemented [6]. Participants in this study acknowledged the potential role of technology in the development of knowledge and skills, particularly if they had features that facilitated behaviour that was interactive, integrated, reflective and which allowed feedback. In addition, ICTs allow for the displacement of content away from the classroom, freeing up time for interactive engagement with other students and the teacher [6]. When combined with the possibility for enhancing content with rich media, ICTs were acknowledged to have a potentially powerful role to play in the development of attributes relevant for clinical practice. However, panelists also discouraged the use of ICTs for it’s own sake, suggesting that a sound pedagogical teaching strategy must drive and support the implementation of technology in teaching practice, echoing the suggestions of other studies in this domain [9]. In addition, technology in teaching needs to be easy to use, and must be perceived by students to have value, if they are to engage with it [10]. For technology-mediated teaching to be effective, it must facilitate communication and reflection in teaching and learning practices that are interactive, flexible and collaborative [8].

Technology can also be used in the creation of online, collaborative spaces that encourage sharing and discussion of clinical encounters and ethical dilemmas. In addition, blended learning approaches have been demonstrated to encourage “flexibility, reflection, interpersonal and teamwork skill development, motivation, and collaborative learning environments - resulting in deep and meaningful understandings” [6]. ICTs may also provide an alternative where face-to-face contact is not possible e.g. students working alone in remote areas. However, panelists also suggested that the use of ICTs in the sharing and discussion of ethical challenges may be best supported with face-to-face reflection and feedback immediately after the clinical encounter.

## Conclusions

It is clear that there are changing conceptions of the knowledge, skills and attitudes required for professional practice, which shift the focus from the products of learning to the process of learning. As clinical educators, we must move beyond describing our students in terms of things they should know and be able to do, and should develop teaching strategies that facilitate a state of professional “being”. We should use teaching practices that integrate knowledge from different curricular domains, that are interactive rather than transmissive, and should accommodate guided, reflective activities that include feedback as part of the curriculum. Technology-mediated instruction does have the potential to change the teaching and learning practices that aim to develop healthcare students who are better equipped to deal with the complexity of clinical practice. However, if we choose to integrate technology into teaching practices that are guided by these principles, then our choices of technological tools should reflect considered outcomes that are framed in the context of what we want our students to be, rather than what we want them to know and to do. Finally, the specific technologies we choose to integrate are less important than the teaching and learning environments we create.

### Limitations and bias

The study has certain limitations and inherent selection bias, including the fact that panel participants were selected by the researcher. Unlike a traditional Delphi study, there was only a limited opportunity for participants to review their responses in summary. However, since there was no aim of determining consensus, this is unlikely to have affected the outcome of the study. Finally, these results and conclusions are most likely highly context-dependent, because of the nature of qualitative research, blended learning, and selection bias.

## Endnotes

^a^ PPD – Personal and Professional Development.

^b^ In the “flipped classroom” students are expected to “consume content in their own time i.e. at home, while homework assignments are completed in class. This gives the teacher the opportunity to spend more time engaging with students, rather than covering content [21].

^c^ f2f: face-to-face.

## Competing interests

The authors declare that they have no competing interests.

## Authors’ contributions

MR carried out the survey design, data acquisition, analysis and interpretation, and major drafting of the paper. JF gave substantive input into the study method, results and discussion sections. VB gave substantive input into development of the study background and discussion sections. All three authors made substantive contributions to the conception and design of the study, engaged in critical review of the final draft, and approved the submission of the paper.

## Authors’ information

MR is a PhD student and lecturer in the Department of Physiotherapy at the University of the Western Cape, and is currently involved in a process of curriculum development. The larger research project is looking at the use of technology-mediated teaching and learning practices in clinical education at an undergraduate level. VB and JF are the project supervisors.

## Pre-publication history

The pre-publication history for this paper can be accessed here:

http://www.biomedcentral.com/1472-6920/13/51/prepub
